# Is LRP2 Involved in Leptin Transport over the Blood-Brain Barrier and Development of Obesity?

**DOI:** 10.3390/ijms22094998

**Published:** 2021-05-08

**Authors:** Elvira S. Sandin, Julica Folberth, Helge Müller-Fielitz, Claus U. Pietrzik, Elisabeth Herold, Thomas E. Willnow, Paul T. Pfluger, Ruben Nogueiras, Vincent Prevot, Thomas Krey, Markus Schwaninger

**Affiliations:** 1 Institute for Experimental and Clinical Pharmacology and Toxicology, Center of Brain, Behavior and Metabolism, University of Lübeck, 23562 Lübeck, Germany; elvira.sandin@gmail.com (E.S.S.); j.folberth@uni-luebeck.de (J.F.); helge.muellerfielitz@uni-luebeck.de (H.M.-F.); 2 Institute for Pathobiochemistry, University Medical Center of Johannes Gutenberg University Mainz, 55099 Mainz, Germany; pietrzik@uni-mainz.de; 3 Institute of Biochemistry, Center of Structural and Cell Biology in Medicine, University of Lübeck, 23562 Lübeck, Germany; elisabeth.herold@uni-luebeck.de (E.H.); krey@biochem.uni-luebeck.de (T.K.); 4 Molecular Cardiovascular Research, Max-Delbrueck-Center for Molecular Medicine, 13092 Berlin, Germany; willnow@mdc-berlin.de; 5 Research Unit Neurobiology of Diabetes, Helmholtz Zentrum München and Technical University Munich, 85764 Neuherberg, Germany; paul.pfluger@helmholtz-muenchen.de; 6 CIMUS, Universidade de Santiago de Compostela-Instituto de Investigación Sanitaria, 15782 Santiago de Compostela, Spain; ruben.nogueiras@usc.es; 7 CIBER Fisiopatología de la Obesidad y Nutrición (CIBERobn), 15782 Santiago de Compostela, Spain; 8 Lille Neuroscience & Cognition, Laboratory of Development and Plasticity of the Neuroendocrine Brain, Inserm, CHU Lille, University Lille, UMR-S1172, EGID, DISTALZ, F-59000 Lille, France; vincent.prevot@inserm.fr; 9 German Center for Infection Research (DZIF), Partner Site Hamburg-Lübeck-Borstel-Riems, 22607 Hamburg, Germany; 10 Excellence Cluster 2155 RESIST, Hannover Medical School, 30625 Hannover, Germany; 11 Centre for Structural Systems Biology (CSSB), 22607 Hamburg, Germany; 12 Institute of Virology, Hannover Medical School, 30625 Hannover, Germany

**Keywords:** leptin, blood-brain barrier, LRP2, ERK1/2, gaussia luciferase, fusion protein

## Abstract

The mechanisms underlying the transport of leptin into the brain are still largely unclear. While the leptin receptor has been implicated in the transport process, recent evidence has suggested an additional role of LRP2 (megalin). To evaluate the function of LRP2 for leptin transport across the blood-brain barrier (BBB), we developed a novel leptin-luciferase fusion protein (pLG), which stimulated leptin signaling and was transported in an in vitro BBB model based on porcine endothelial cells. The LRP inhibitor RAP did not affect leptin transport, arguing against a role of LRP2. In line with this, the selective deletion of LRP2 in brain endothelial cells and epithelial cells of the choroid plexus did not influence bodyweight, body composition, food intake, or energy expenditure of mice. These findings suggest that LRP2 at the BBB is not involved in the transport of leptin into the brain, nor in the development of obesity as has previously been described.

## 1. Introduction

Secreted from adipocytes, leptin binds to its receptor (LepR) in various tissues to control several functions in the human body [1]. Most notably, leptin regulates feeding and reward-seeking behavior, as well as memory by binding to several regions in the brain [2,3,4,5]. In the hypothalamus, the primary region regulating appetite and feeding behavior, leptin binds to the long isoform of the LepR (LepRb), which leads foremost to the phosphorylation of signal transducer and activator of transcription 3 (STAT3) [6,7]. In the arcuate nucleus (Arc) of the hypothalamus, this signal transduction reduces food intake via a complex regulation of feeding behavior through the proopiomelanocortin (POMC) and agouti-related protein (AgRP) positive neurons [8,9,10]. Some studies have shown that leptin also alters energy expenditure, although this has recently come into dispute [6,11,12]. Perhaps unsurprisingly, the discovery of leptin and its unequivocal importance for obesity, a global health threat [11,12], led to a strong focus on the weight-regulating and potential anorexic effect of leptin. However, it was soon discovered that administering leptin to obese individuals to reduce appetite was unsuccessful because obesity is associated with a resistance to leptin as a result of excessive adipose deposits and subsequent excessive leptin levels [13,14]. Research on diet-induced obese (DIO) mice suggests that this leptin resistance is primarily peripheral, since leptin administered directly into the brain does exert its appetite-reducing effect in DIO mice [15,16].

The findings that DIO mice exhibit peripheral, but not central, leptin resistance may be due to an impaired delivery of leptin into the brain. Leptin has to cross the blood-brain barrier (BBB) to reach its primary site of action [17]. So far, the molecular mechanisms underlying transport of leptin across the BBB remains controversial. While there is strong evidence for the involvement of the short isoforms of LepR [18,19,20,21,22], recent evidence has illustrated that the LepR is likely not the only receptor involved in leptin transport across the BBB [3,23]. Some attention has also recently been given to non-STAT3-related signaling by LepR [24]. LepR is expressed at low levels in brain endothelial cells and the principal isoforms expressed there are short, lacking the long intracellular tail required for STAT3 signaling [20,25]. Despite lacking the long intracellular tail, some of the short isoforms retain signaling capacity [20,26,27]. The activation (phosphorylation) of p42/44 mitogen-activated protein kinase (ERK1/2) may be of particular interest at the BBB since its phosphorylation has been found to promote transport across tanycytes at the third ventricle [28]. 

Another potential transporter of leptin is the low density lipoprotein-related protein 2 (LRP2). LRP2, also known as megalin, is a large endocytic receptor that has been implicated in the transport of many proteins [29]. LRP2 is expressed in the BBB and in the choroid plexus [26,27,30,31]. It binds and transports leptin across epithelial barriers, including the blood-cerebrospinal fluid (CSF) barrier [30,32]. Mice with an endothelial specific knockout of LRP2 exhibited obesity when fed normal chow diet [33]. However, this mouse model used a constitutively active Tie2-Cre driver line that is active in all endothelial cells of the body and in hematopoietic cells [34,35]. This raises the question whether the alterations in bodyweight in the Tie2-Cre driver line are caused by LRP2 in the BBB or elsewhere. In this study, we aim to re-evaluate this question by deleting LRP2 specifically in BBB endothelial and choroid plexus epithelial cells using the tamoxifen-inducible Slco1c1-CreER^T2^ driver line. This mouse line is specific for the CNS [36].

To trace leptin in transport studies across BBB endothelial cells, we fused leptin to Gaussia luciferase (pLG). With this tool, we traced leptin transport across an in vitro porcine BBB model, which is widely regarded as one of the most valid BBB models [37]. To characterize the transport, we applied the LRP antagonist receptor-associated protein (RAP) [38] and an ERK1/2 signaling inhibitor. The in vitro data as well as the phenotype of mice deficient of LRP2 in brain endothelial and epithelial cells provided no evidence for the involvement of LRP2 in brain barrier cells in mediating the leptin effects.

## 2. Results

### 2.1. Novel Leptin-Gaussia Luciferase Fusion Protein Is Taken Up by A BBB Model and Activates the LepRb

**Figure 1:**
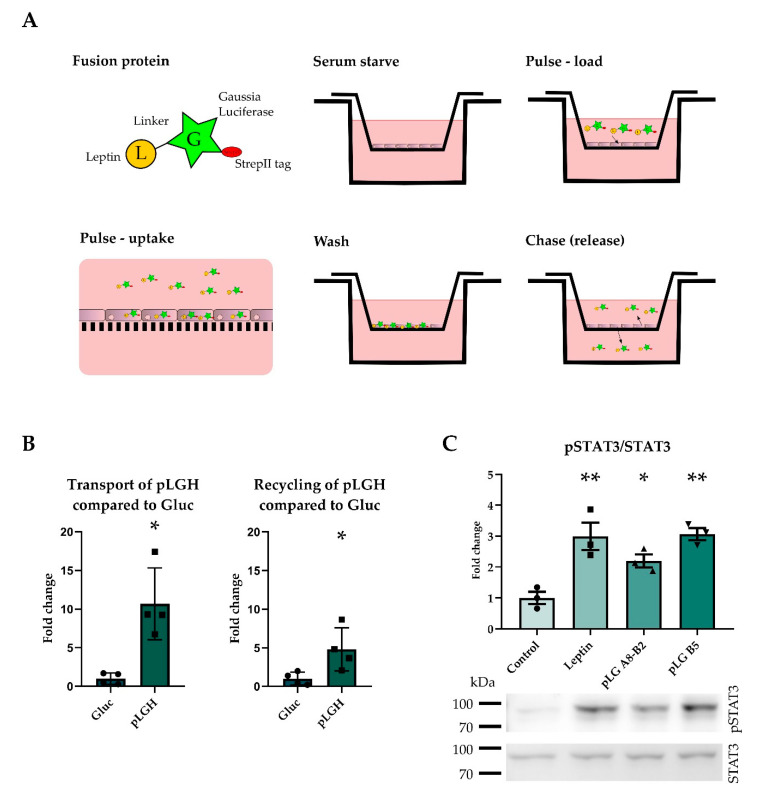
The leptin-Gaussia luciferase fusion protein activated the LepR and was taken up into brain endothelial cells. The pulse chase assay is depicted, in which the protein is taken up by the cells during the pulse phase, excessive protein is washed off, and the cells are allowed to release the protein during the chase phase. Upper chamber release is reported as recycling and lower chamber release as transport (**A**). The his-tagged fusion protein pLGH was transported and recycled to a higher degree than Gluc (**B**), indicating that the cells take up the pLGH protein (*n* = 4). pLG activated the leptin receptor, as shown by STAT3 phosphorylation in murine leptin receptor overexpressing CHO cells (**C**) (*n* = 3). Results are presented as mean ± SEM * *p* < 0.05 ** *p* < 0.01.

As a model of the BBB, we used porcine brain endothelial (pBCEC) cells in a two chamber insert system (Figure 1A). To enhance the barrier properties, we transiently co-cultured the pBCEC with immortalized rat astrocyte cell line CTX-TNA2 for 4 days and treated them with hydrocortisone, cAMP, and a cAMP phosphodiesterase inhibitor. The trans-endothelial electrical resistance (TER) of cells were typically in the range of 150 Ω·cm² as measured by cellZscope, while we obtained much lower TER values with mouse brain endothelial cells (data not shown). 

In pulse-chase experiments of pBCEC, we added Gaussia luciferase or the fusion protein porcine leptin-Gaussia luciferase to the upper compartment and measured Gaussia activity after a chase in the upper (recycling) or lower compartment (transport, Figure 1A). The fusion protein was based on previous successful attempts to fuse proteins to Gaussia luciferase in order to analyze transport or receptor binding [39,40]. The fusion protein porcine leptin-Gaussia luciferase with a his tag (pLGH) showed more transport and recycling than the molar equivalent amount of Gaussia luciferase (Gluc) (Figure 1B). In order to improve the quantity and purity of the fusion protein, the his tag was exchanged for a strepII tag and the fusion protein (pLG) was expressed in insect cells and purified (Figure S1).

To test the leptin activity of pLG at LepR, we expressed the long isoform LepRb in Chinese hamster ovarian (CHO) cells. Two fractions (A8-B2 and B5, Figure 1C and Figure S1) of the purified fusion protein pLG stimulated STAT3 phosphorylation to a similar extent as leptin, demonstrating that the C-terminal Gaussia does not interfere with leptin’s capacity to bind to LepR.

### 2.2. Recombinant Leptin, Inhibition of Phosporylation of ERK, but Not LRP2 Binding Sites, Reduces pLG Transport and Recycling in A Pulse-Chase Assay

**Figure 2:**
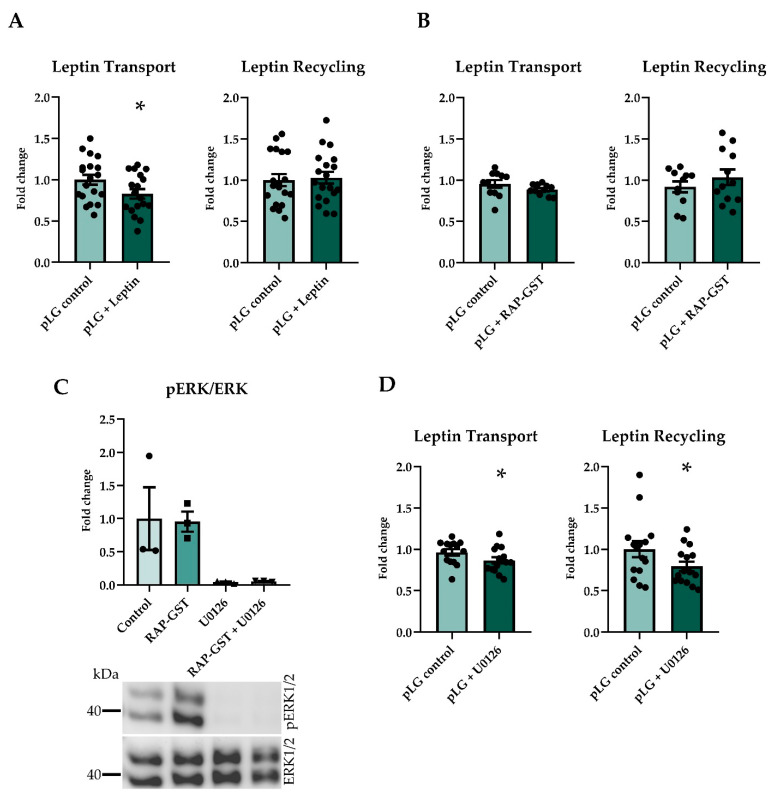
Pulse chase assays using different inhibitors and co-treatments. Pretreatment and co-treatment with recombinant porcine leptin reduced transport but not recycling (**A**) (*n* = 18–19). RAP-GST, an LRP antagonist, did not inhibit the transport or recycling of leptin (**B**) (*n* = 11–12). U0126 abolished the phosphorylation of ERK1/2, also when co-treated with RAP-GST, and RAP-GST did not influence the phosphorylation of ERK1/2 (**C**) (*n* = 3). Inhibiting the phosphorylation of ERK using U0126 significantly reduced transport and recycling of leptin (**D**) (*n* = 14–15). Results are presented as mean ± SEM * *p* < 0.05.

The transport but not the recycling of pLG was significantly reduced by recombinant porcine leptin (200 nM, Figure 2A). pBCEC expressed *Lrp2* (data not shown). However, the transport and recycling of pLG was unchanged by the LRP antagonist RAP-GST (Figure 2B). The ERK signaling inhibitor U0126 abolished the phosphorylation of ERK1/2 in porcine brain endothelial cells, but the treatment with RAP-GST did not affect the pERK1/2 levels (Figure 2C). Treatment with U0126 significantly reduced the leptin transport and recycling (Figure 2D).

### 2.3. Deficiency of LRP2 in the Choroid Plexus and Endothelial Cells of the Blood-Brain Barrier Does Not Alter Body Composition or Metabolism in Mice

**Figure 3:**
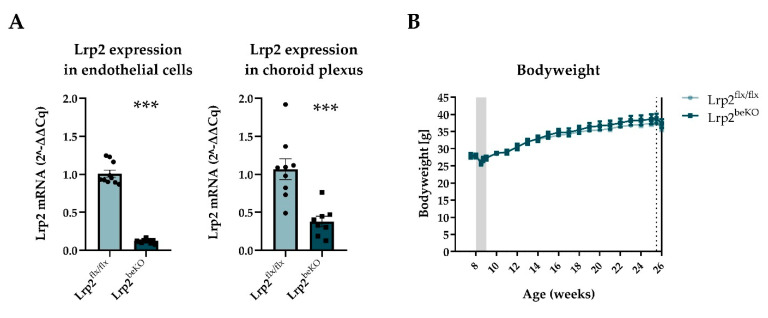
Endothelial cell and choroid plexus LRP2 reduction but no alteration of bodyweight in *Lrp2* brain endothelial knockout mice (Lrp2^beKO^) compared to Lrp2 floxed littermates (Lrp2^flx/flx^). Lrp2 was successfully deleted in both brain endothelial cells and choroid plexus (**A**) *n* = 8–10. No change in body weight was observed between Lrp2^beKO^ mice and Lrp2^flx/flx^ mice over time. Shaded area indicates tamoxifen injection days, dotted line indicates body composition measurement, and whole line indicates start of calorimetry (**B**) *n* = 10. Results are presented as mean ± SEM *** *p* < 0.001.

To delete *Lrp2* specifically in brain endothelial and epithelial cells we used the inducible Slco1c1-CreER^T2^ mouse line crossed with Lrp2^flx/flx^ animals (Lrp2^beKO^) [36,41]. The efficiency of the *Lrp2* knockout in Lrp2^beKO^ mice is illustrated by an 88.6% reduction of *Lrp2* mRNA in primary brain endothelial cells and 68.9% reduction in the choroid plexus compared to Lrp2^flx/flx^ mice (Figure 3A). In the plexus, recombination is specific for epithelial cells and does not involve stromal cells [36], explaining the incomplete deletion of *Lrp2* in extracts of the whole tissue. Bodyweight increased significantly with age but no difference in bodyweight between genotypes was observed over the lifetime of the mice (Figure 3B). Correspondingly, there was no difference in body composition (Figure S2A) or food and water intake (Figure S2B,C). Respiratory exchange ratios and energy expenditure fluctuated over the time of day, as mice are more active during the dark phase, but were also not altered by the *Lrp2* knockout (Figure S2D,E).

## 3. Discussion

Leptin is secreted by the adipose tissue and enters the brain to exert its main effects on metabolism. Multiple lines of evidence suggest that the passage through the BBB is a limiting step in leptin’s ability to reach its site of action [17]. Therefore, the mechanisms by which leptin reaches the brain have received extensive attention. One of the potential transporters of leptin is LRP2, which is expressed in brain endothelial and epithelial cells [26,27,30,31]. Indeed, LRP2 is able to transport leptin in epithelial cells [30,32]. Deleting LRP2 in endothelial cells with the help of the Tie2-Cre mouse line [34] led to an increased bodyweight, in line with the concept that leptin utilizes LRP2 in the BBB to reach its site of action [33]. However, when the Tie2-Cre; Lrp2^flx/flx^ animals EMD mice in [33,42] were challenged with a high-fat diet, they did not develop obesity as wild-type animals did [42], arguing against a role of LRP2 as leptin transporter in the BBB. Interpretation of the phenotype of Tie2-Cre; Lrp2^flx/flx^ mice is confounded by the activity of the driver line in endothelial cells of all tissues, endocardial cells, and the hematopoietic cell line. *Lrp2* is expressed in endocardial cells, myeloid cells, in developing endothelial cells, and is involved in hematopoiesis [43,44,45], suggesting that the phenotype of Tie2-Cre; Lrp2^flx/flx^ mice may be due to effects generated outside of the BBB.

Therefore, we herein re-evaluated the role of LRP2 in the transport of leptin across brain endothelial cells. To facilitate the detection and quantification of leptin, we fused it with Gaussia luciferase. The fusion protein retained its activity at the leptin receptor, and its transport could partially be inhibited by co-treatment with recombinant leptin. The transport of leptin was reduced when we inhibited ERK signaling. This is in line with a previous study showing that the transport of leptin by tanycytes is enhanced by ERK activation [28]. In contrast, the RAP protein, a natural inhibitor of ligand binding to LRP2, had no effect on leptin transport or recycling suggesting that LRP2 is not instrumental for the transport of leptin by brain endothelial cells, at least in the porcine model that we have investigated. This notion was supported by the phenotype of mice with a selective LRP2 deficiency in brain endothelial cells. With the genetic strategy, we achieved an 88.6% reduction of *Lrp2* mRNA levels in brain endothelial cells and a 68.9% reduction in the choroid plexus. However, in contrast to previous studies using a Tie2-Cre driver line [33], the knockout of *Lrp2* in brain endothelial and epithelial cells of mice did not result in a difference in bodyweight, body composition, food intake, or energy expenditure. This is especially striking since we herein used the same floxed mouse line that was crossed with this Tie2-Cre driver line, and a similar reduction of LRP2 expression has been reported with the Tie2-Cre driver line [41,46]. The findings showing that Tie2-Cre; Lrp2^flx^/^flx^ mice are protected against DIO, suggest that the role of LRP2 in endothelial cells is complex. It also indicates that the global endothelial expression of Tie2 might confound and limit the interpretations that can be made with regards to the BBB. It is also important to consider that our model is tamoxifen inducible and the Tie2-Cre model uses a constitutive Cre recombinase. One major benefit of tamoxifen inducible knockout mouse models is that they circumvent a potential influence during development and enable the temporal control of the knockout. However, tamoxifen treatment itself reduces bodyweight during and directly after treatment, which is also visible in our bodyweight data. This is due to the stress of injection and handling and to the pharmacological properties of the compound. Tamoxifen is able to influence body fat and bodyweight for up to 5 weeks after treatment [47], highlighting the importance of a recovery period. Tamoxifen is a selective estrogen receptor modulator with different impacts on female and male animals. In line with this mode of action, tamoxifen treatment induced white adipose tissue browning in female but not in male mice [48]. Modulation of estrogen receptors may directly influence energy homeostasis [49]. A recent study showed that tamoxifen treatment can increase leptin sensitivity in ovariectomized female mice [50]. In the calorimetric experiments of the present study, the recovery period after tamoxifen treatment was approximately 18 weeks, and only male mice were used for calorimetric and body composition measurements. Thus, tamoxifen is unlikely to have influenced these measurements in our mice.

The current study suggests that LRP2 in the BBB is not involved in leptin transport in vitro or obesity in mice under normal chow diet feeding conditions. In line with this concept, obesity is not a feature of the autosomal recessive Donnai-Barrow syndrome that is caused by inactivating bi-allelic mutations in LRP2 [51]. However, our findings do not exclude the involvement of LRP2 during DIO conditions. Notably, the deletion of LepR in the BBB only became phenotypically significant during DIO [3]. We can also not exclude that LRP2, by binding and transporting Abeta, has indirect effects on leptin penetration into the brain [46]. Since we cannot specifically conclude that leptin transport is not altered in vivo, future studies should strive to further elucidate possible mechanisms of leptin resistance and the involvement of LRP2 in leptin transport by exploring leptin plasma and brain levels, as well as basal leptin transport into the brain, in the herein presented LRP2 BBB knockout model. Nonetheless, in light of recent results using the Tie2-Cre; Lrp2^flx^/^flx^ mice, our preliminary data using Slco1c1-CreER^T2^; Lrp2^flx^/^flx^ mice indicate that LRP2 at the BBB is not specifically involved in the development of obesity. Rather, LepR and intracellular signaling cascades such as phosphorylation of ERK1/2 in tanycytes of the mediobasal hypothalamus and endothelial cells at the BBB seem to be principal regulators of transport leptin across brain barriers and obesity [3,28].

## 4. Materials and Methods

### 4.1. Leptin-Gaussia Luciferase Fusion Protein (pLG) and Its Precursor pLGH

A custom designed mammalian expression plasmid containing the pLG construct was originally designed and ordered from Vectorbuilder (Chicago, IL, USA). This plasmid contained the full sequence of porcine leptin excluding the stop codon at the end of the sequence, followed by a (Gly4Ser)2 linker, and ending with the sequence of Gaussia luciferase excluding its secretory sequence and a 6His tag before the stop sequence, we called this pLGH. The pLG construct used for the pulse chase assays in this paper was transferred from the pLGH mammalian expression plasmid into an insect cell expression plasmid (pT627) by IVA cloning as previously described [52]. The pLG construct differed only in that the leptin secretory sequence and the 6His tag are excluded, since the pT627 receiver plasmid contained a 2x Strep II tag and an insect secretory sequence [53]. The resulting plasmid produced a protein, porcine Leptin-Gaussia luciferase, which is a 38.6 kDa protein (Figure S1). This protein was purified as previously described [54] (see also Supplementary Materials and Methods).

### 4.2. LepRb Overexpression in CHO Cells and pLG Treatment

The CHO cells were transfected with the plasmid 426-pCI-HA-mOBRb encoding the mouse LepRb [55] but were not treated for stabilization. Instead, the cells were trypsinised after 48 h and split into 6-well plates for treatment. One day after seeding, the cells were serum starved for 3 h and then treated with 10 nM recombinant porcine leptin (ProSpec, Rehvot, Israel) or the pLG fraction in equimolar concentrations for 30 min. The cells were then washed once with PBS and lysed with NP40 lysis buffer (1% Igepal, 150 mM NaCl, and 50 mM Tris (pH 8) in PBS).

### 4.3. Primary Porcine Brain Endothelial Cell Culture (pBCEC)

Brains from Piétrain/Swabian-Hall swine were generously provided by the Robert Prösch slaughterhouse in Krummesse, Germany. All cell culture and dissection was performed under a Laminar-Flow workbench and cultured at 37 °C with 5% CO_2_. 

The brains were transported in cold transport medium (0.3 µg/mL amphotericin B (Biochrom GmbH, Berlin, Germany) and 1% pen/strep (Sigma-Aldrich, St. Louis, MO, USA) in PBS (Biowest, Nuaillé, France)) from the slaughterhouse to the laboratory. Each hemisphere was individually removed from the transport container and dissected in fresh sterile transport medium under the laminar flow hood. After removal of the meninges, the grey matter was peeled off using a scalpel and directly transferred into a 50-mL Falcon tube containing dispase II (Roche Diagnostics GmbH, Mannheim, Germany) (23 mL, 10 U/mL, room temperature). The tube was filled up to match the volume of dispase, e.g., 45–46 mL total volume. The tissue was triturated with a 25-mL and then with a 10-mL pipette 10 times and then digested by shaking on a platform (37 °C, 150 rpm, 70 min).

The tissue suspension was transferred onto a discontinuous density gradient with final content of 40% tissue, 24% percol (GE Healthcare Life Sciences, Little Chalfon, UK) and 36% DMEM (PAN-Biotech GmbH, Aidenbach, Germany). The myelin and vessel fragments were then separated by centrifugation at 2890× *g* at 4 °C for 30 min, without break stop. The uppermost myelin layer was carefully removed by aspiration, and the vessel fragments were collected and transferred into a fresh 50-mL tube. The vessel fragments were further digested at 37 °C in collagenase type II (1.67 mg/mL, Biochrom), shaking at 150 rpm for 21 min. For every 1 mL of collagenase, 3.3 mL DMEM and 3.3 µL 10 µg/µL DNase I (Roche) was added and further incubated for 5 min. The cells were then filtered through a 70-μm cell strainer and pelleted by centrifugation (10 min, 4 °C, 1000× *g*). The pellet was resuspended in DMEM F12 (PAN-Biotech GmbH) containing 10% bovine plasma-derived serum (PDS) (FirstLink, Wolverhampton, UK) and 1% pen/strep. The cells were then seeded into T75 flasks pre-coated with collagen h placenta (Sigma-Aldrich, St. Louis, MO, USA), and cultured for 24 h with 2.5 µg/mL amphotericin 10 µg/mL gentamicin (Gibco by Life Technologies Limited, Carlsbad, CA, USA), and 4 µg/mL puromycin. The cells were then further cultured for 24 h with 4 µg/mL puromycin. 

The pBCEC were then split by trypsinization and seeded into collagen h placenta precoated Greiner Bio-One thincert 24-well cell culture inserts translucent membrane with a pore size of 0.4 µm (Greiner Bio-One International GmbH, Kremsmünster, Austria) at a density of 15–20,000 cells/insert. On the same day, CTX-TNA2 immortalised rat astrocytes were seeded into matching 24-well cell culture plates at a density of 62,500/well in DMEM F12 with 5% FCS [56]. After allowing the CTX-TNA2 to attach over-night, the pBCEC inserts were transferred into the CTX-TNA2 plate for co-culture. 

After 4 days of co-culture, the pBCEC were transferred into the cellZscope (nanoAnalytics GmbH, Münster, Germany) for trans-endothelial electrical resistance (TER) and capacitance measurements. At this point, the media was changed to DMEM F12 with 10% PDS in the insert and serum-free DMEM F12 in the well to further polarize the cells. When capacitance had stabilized below 1, after approximately 1–3 days in the cellZscope, the media was changed to DMEM F12 with 5% PDS, 500 nM hydrocortisone, 17.5 µM RO20-1724, and 250 µM pCPT-cAMP (all, Sigma-Aldrich) in the well for 24 h. Directly following this, the pBCEC were used for pulse chase assays.

### 4.4. Pulse-Chase Assay

The pBCEC were transferred from the cellZscope to 24-well plates (Greiner Bio-One), the media was changed to serum free DMEM F12, and cells were serum starved for 3 h. Fresh 24-well plates were loaded with 500 µL/well and pre-warmed at 37 °C with 5% CO_2_ for both the pulse and chase phase. For all treatments and co-treatments, the corresponding vehicles and buffers were included in the simultaneous treatments and/or control treatment. The pre-treatment start varied depending on the compound. For leptin, the cells were serum starved for 2 h and then porcine leptin (200 nM, ProSpec-Tany TechnoGene Ltd., Rehovot, Israel) was added to the media in the insert (150 µL) and pre-treated for 1 h. For RAP-GST or U0126 (Sigma-Aldrich), the cells were serum starved for 150 min and then RAP-GST (500 nM) or U0126 (10 µM) was added to the media in the upper compartment (150 µL) and pre-treated for 30 min. For the control condition, the corresponding buffer was added at the corresponding pre-treatment time point. This resulted in a total serum starvation for 3 h for all treatments. The media was then aspirated from the insert and well, and 150 µL DMEM F12 containing 2 nM pLG fusion protein (pLG control) or 2 nM pLG fusion protein and 200 nM leptin (pLG + Leptin), or 500 nM RAP-GST (pLG + RAP-GST), or 10 µM U0126 (pLG + U0126) was loaded into the insert, and the inserts were transferred into pre-warmed 24-well plates. The pulse (uptake) with pLG was incubated for 30 min at 37 °C with 5% CO_2_. 

The media from the inserts and wells was aspirated and any residue still on the insert was carefully removed. Each insert was then washed once by adding 200 µL cold DMEM F12 to the insert. Each insert was individually aspirated and completely submerged in cold DMEM F12 three times, on ice. Any remaining media in or on the insert was carefully removed and 150 µL 37 °C DMEM F12 was added to the insert, and then the insert was transferred onto the pre-warmed 24-well plate. The chase (release) phase (Figure 1A) was incubated for 60 min at 37 °C with 5% CO_2_. Then, the plates were transferred onto ice and 20 µL was collected in duplicates from the insert (recycling) and the well (transport) of each sample.

Directly after sampling, luciferase activity was measured in black 96-well plates (Corning GmbH, Mannheim, Germany), using 20 µL samples in duplicates and 80 µL substrate consisting of 20 µM coelenterazine (NanoLight^®^ Technologies, Prolume Ltd., Pinetop-Lakeside, AZ, USA) in PBS with 0.001% Tween 20. The raw light units (RLU) were measured without any filter and with a gain setting of 4095. The RLU were measured every second for 7 s, and the peak of the RLU was selected for analysis; this peak was exclusively the first measurement after injection. Fold change from pLG control was calculated based on this value.

### 4.5. Inhibition of Phosphorylation of ERK with U0126 in pBCEC

pBCEC were cultured as described above, but were seeded into 6-well inserts (75.000–100.000/insert). U0126 and RAP-GST treatment was performed as described in the pulse-chase, but the cells were not treated with pLG and were washed once with PBS and lysed with NP40 lysis buffer directly after the 30 min pulse phase.

### 4.6. RNA Extraction and qPCR

RNA was extracted from cells using the nucleospin RNA extraction kit (Macherey-Nagel, Düren, Germany) according to the kit protocol. The concentration of RNA was estimated using Nanodrop (Thermo Fisher Scientific, Waltham, MA, USA). Samples were then diluted to 500 ng (endothelial cells) or 300 ng (choroid plexus) in 35 μL DEPC water, and a mastermix (10 μL 5× MMLV buffer, 2 μL 25 mM dNTP mix, 1 μL random hexamer, 1 μL MMLV reverse transcriptase, and 1 μL Rnasin; all Promega, Madison, WI, USA) was added to each sample. The tubes were incubated for 10 min at room temperature, followed by 90 min at 37 °C, and then stored at −20 °C until use. 

The qPCR was performed using half reaction of the Platinum^®^ SYBR^®^ Green qPCR SuperMix (Invitrogen, Carlsbad, CA, USA) as per the kit protocol. The reactions were measured on the Roche LightCycler^®^ 96 System. The forward primer (5′-TGA CCT CCC CAA ATG CAA GT-3′) and the reverse primer (5′-CAG AAG AGC CAT TGT CGT CC-3′) of *Lrp2* (NM_001081088.2) are located on the exon spanning junctions between the 71st–72nd and 72nd–73rd exon. LoxP sites in our knockout model flank exon 71 and exon 73, which code for the membrane anchoring part of the LRP2 [41].

### 4.7. Animals

Male or female mice with an inducible deletion of *Lrp2* in brain endothelial and epithelial cells (Lrp2^beKO^) were generated by crossing Lrp2^flx/flx^ [41] and Slco1c1-CreER^T2^ [36] animals. Male and female mice were used in the cohort to determine the efficacy of the knockout (Figure 3A); the sex of the mice were evenly distributed between knockout and control. A second cohort of only male mice was used for the bodyweight, body composition, and calorimetric measurements (Figure 3B and Figure S2A–E). The knockout was induced by tamoxifen injection when the animals were 6–9 weeks of age. Control mice (Lrp2^flx/flx^) received the same tamoxifen treatment. The tamoxifen (Sigma-Aldrich) was dissolved to a concentration of 20 µg/µL and injected intraperitoneally with 50 µg/g of bodyweight every 12 h for 5 consecutive days. The animals were housed in groups of 2–5 animals in individually ventilated cages (IVC) on a 12 h light/dark cycle, at 23 °C ± 1 °C. The genotypes in each cage were balanced to the greatest extent possible. The animals were fed a normal chow diet at libitum. The animals were kept under these conditions until they were placed into the calorimetric system. 

#### 4.7.1. Indirect Calorimetry and Body Composition Measurements

For indirect calorimetry and body composition measurements, we used only male mice. Body composition (gram) of the animals was assessed using nuclear magnetic resonance (NMR) in the Bruker minispec LF110 (Bruker, Billerica, MA, United States). The body composition percentage was then calculated based on the values produced by the NMR and the bodyweight of each animal directly before the measurement. Three days after body composition measurement, the animals were placed into individual cages for the indirect calorimetric measurements. The mice indirect calorimetry was performed with the PhenoMaster System (TSE, Germany); measurement of O_2_ and CO_2_ was taken every 30 min for the determination of respiratory exchange ratio (RER) and energy expenditure (EE) for 7 days. Food and water intake was measured continuously and automatically as the animals ate or drank. Since stress and novelty factors strongly influence these parameters in the beginning of the measurement, only the measurements of the last 3 days have been used for analysis because they are the most stable. EE and RER were calculated as reported previously [57].

#### 4.7.2. Choroid Plexus Extraction and Primary Mouse Brain Endothelial Cell Culture

The choroid plexus was extracted from both lateral ventricles and lysed with RNA nucleospin R1 lysis buffer. The brains were then directly transferred to the cell culture for brain endothelial cell culture. Primary mouse brain endothelial cells were cultured as reported previously [58]. Once the mouse brain endothelial cells were confluent, after 4–5 days in culture, they were lysed with nucleospin RNA R1 lysis buffer and RNA was extracted as described above. 

### 4.8. Statistics

A one way *t*-test was applied (Figure 1B, Figure 2A,B,D and Figure 3A,C–E), unless distribution of variances was found to be significantly different, in which case a Mann–Whitney U test was performed instead. In experiments with more than two conditions, one way ANOVA with a Dunett post-test comparing treatments to the control group was applied (Figure 1C and Figure 2C). Two-way ANOVA (Time × Genotype) was performed on bodyweight development (Figure 3B). Outliers were removed using Grubbs (Alpha = 0.1) test.

## Data Availability

The data are available from the authors upon request.

## Supplementary Materials

The following are available online at https://www.mdpi.com/article/10.3390/ijms22094998/s1: Figure S1: Purification of the pLG fusion protein from pT627 pLG; Figure S2: Indirect calorimetry and body composition of Lrp2beKO compared to Lrp2flx/flx mice. Supplementary methods: Western Blot, pLGH and pLG purification. Table S1. Primary and secondary antibodies used for western blot.
