# Supplementary material for: Is LRP2 Involved in Leptin Transport over the Blood-Brain Barrier and Development of Obesity?
Source: Int J Mol Sci. 2021 May 8;22(9):4998. doi: 10.3390/ijms22094998 (PMC8125945; doi:10.3390/ijms22094998)
Supplement: Supplementary file 1 [file ijms-22-04998-s001.zip › ijms-1187150-supplementary.pdf]

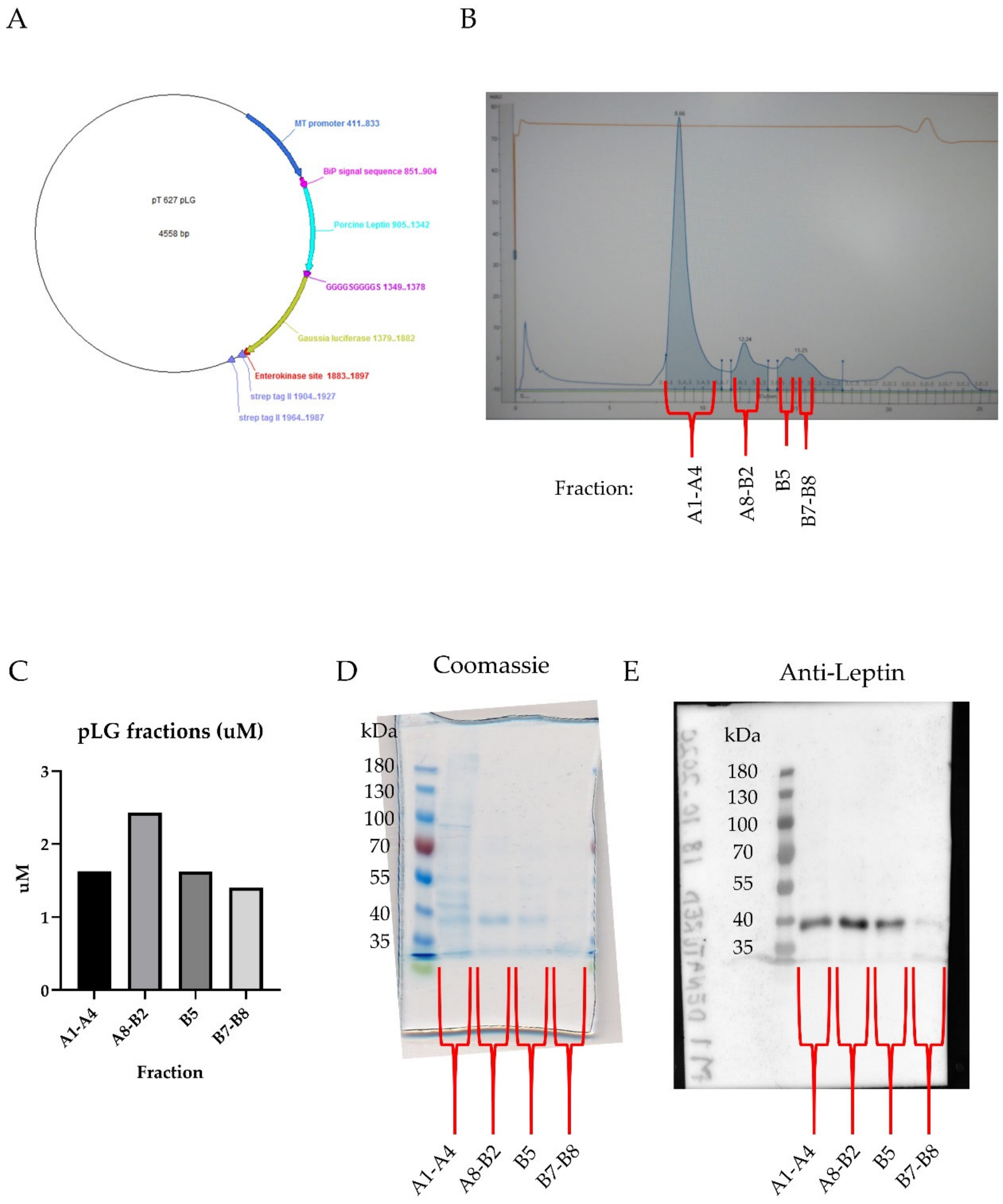

Figure S1. Purification of the pLG fusion protein from pT627 pLG. pLG was purified from the pT627 insect expression plasmid (A). The fractions that resulted (B) all illustrated luciferase activity between 1-2.5uM (C) when plotted against a standard curve of recombinant gaussia luciferase. However, the purity of fraction A1-A4 was compromised as illustrated in the Coomassie stain (D), and the quantity was low in fraction B7-B8 as was observed both in the coommassie (D) and anti-leptin stain (E). For these reasons, fraction A8-B2 and B5 were the fractions that were tested on LepRb overexpressing CHO cells.

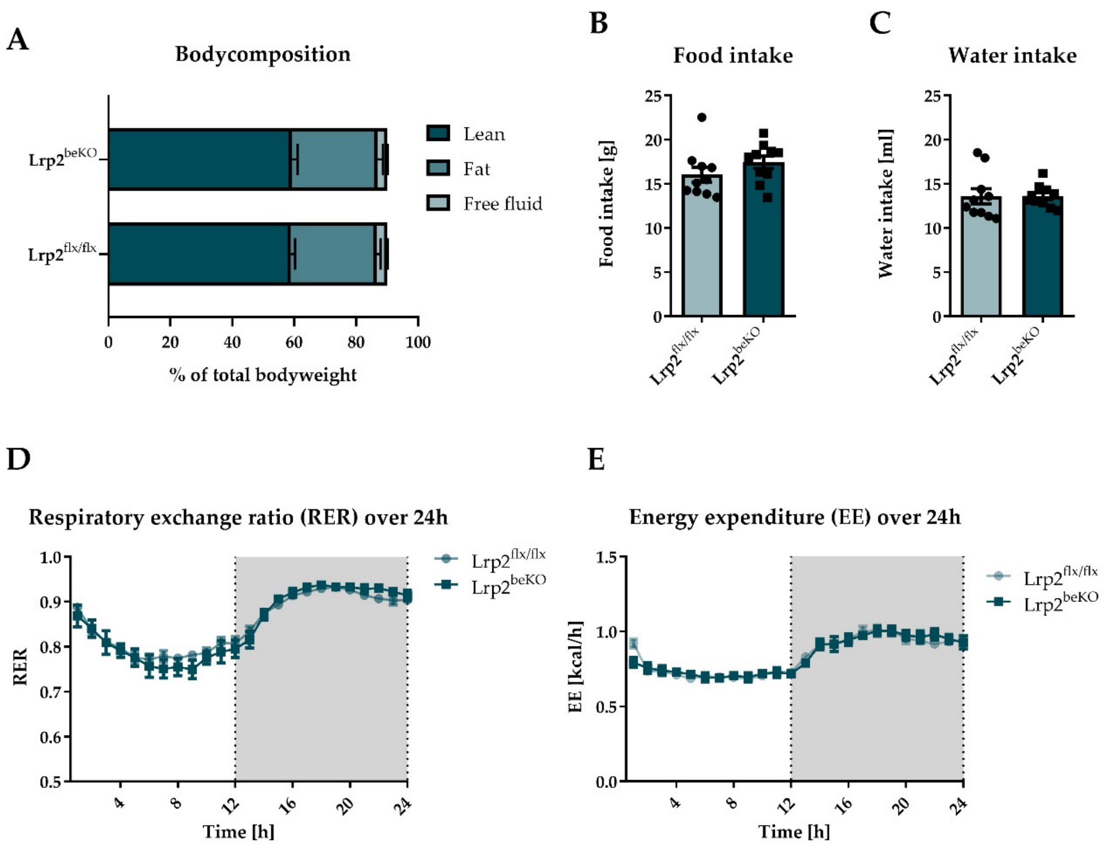

Figure S2. Indirect calorimetry and body composition of Lrp2^{beKO} compared to Lrp2$^{flx/flx}$ mice. No difference was observed in any body composition parameters (A) n=10. Correspondingly, food (B) and water (C) intake were similar in Lrp2^{beKO} and Lrp2$^{flx/flx}$ mice (both n=10). Respiratory exchange ratio (RER, D) and energy expenditure (EE, E) also did not differ between groups, both n=10. Food intake and water intake represent the sum of the last 3 days in calorimetry. RER and EE are the average of each corresponding hour for each corresponding mouse over the last 3 days in calorimetry. Results are presented as mean ±SEM *p<.05 **p<.01 ***p<.001.

Supplementary methods.

1. Expression and purification of pLGH and pLG (Accompanying Figure S1)

CHO cells were cultured in DMEM F12 (PAN-Biotech GmbH, Aidenbach, Germany) with 5 % fetal calf serum (FCS, Sigma-Aldrich, St. Louis, USA) and 1 % penicillin-streptomycin (pen/strep, Sigma-Aldrich). The pLGH mammalian expression plasmid was transfected into CHO grown in 10-cm diameter cell culture plates using calcium phosphate precipitation. Briefly, 400 ng/cm^2 pLGH plasmid in 50 µl sterile water was added to 62.5 µl 2 M $CaCl_2$ and 387.5 µl sterile water. 2xBBS (0.5 ml, 50 mM BES, 280 mM NaCl, 1.5 mM Na_2HPO_4, pH 6.95) was added to the tube. After 3 min the mixture was added to the media and cells were incubated for 24 h at 37°C with 3 % CO_2. The media was then changed, and CO_2 was turned up to 5 %. The cells were incubated another 24 h and then detached with trypsin 0.05 %/EDTA 0.02 % in PBS (PAN-Biotech GmbH) for 2-3 min and seeded into a T75 flask. At this point, the cells were incubated with puromycin (8 µg/ml, Sigma-Aldrich) to stabilize the transfection. The cells were incubated with puromycin, changing the media every 2-3 days. After two weeks the cells were trypsinized as above and seeded into 15x T175 flasks with DMEMF12 containing 2.5 % FCS. Since the pLGH was secreted into the media, the cells were incubated for 48 h and the media collected for purification. The media was centrifuged at 1000 x *g* to pellet any debris. The Hispur Ni-NTA 3 ml spin column kit (Thermo Fisher Scientific, Waltham, USA) was then used with some modification for media extraction. An equal volume of equilibration buffer was added to the media together with 3 ml Ni-NTA beads from the kit, prepared as per kit instructions. The media/buffer was incubated at 10 °C shaking at 150 rpm over-night. The beads were pelleted at 700 x *g* 4 °C and the kit protocol was resumed.

For production in Drosophila Schneider 2 (S2) cells stable transfectants were generated as previously described [55]. Briefly, the pLG expression plasmid was co-transfected with a dominant selectable marker encoding puromycin acetyltransferase using Effectene (Qiagen) according to the manufacterer's recommendation. For protein production an approximate culture volume of 2,4 l was induced with a final concentration of 4 µM $CdCl_2$ at a density of 8 × 10^6 cells per ml. Six days after induction the supernatant was concentrated to a volume of 300 ml and applied to a 8 ml Strep-Tactin XT column according to the manufacterer's recommendation. Subsequently, the protein was further purified by size exclusion chromatography using a Superdex 200 Increase 10/300 column pre-equilibrated with PBS.

2. Western blot

The western blots were performed using 10 % polyacrylamide gels. All samples were blotted on two membranes, one membrane was then used for staining phosphorylated protein and the other for non-phosphorylated protein. The membranes were stained with Ponceau S (Sigma-Aldrich), imaged, and then washed 3x 10min in TBS-T. The membranes were then blocked in blocking buffer (5 % bovine serum albumin (Sigma-Aldrich) in TBS-T) for 90 min. The primary antibodies (Table S1) were incubated cold in blocking buffer over-night on a roller. The membranes were then washed 3x 10 min in TBS-T and the secondary antibody (Table S1) was incubated for 90min diluted in blocking buffer. The membranes were washed 3x 10 min and developed using Enhanced chemiluminescence SuperSignal™ (Thermo Fisher Scientific) according to the kit instructions. The imaging was done on a Fusion Solo S (Vilber Lourmat Sté, Collégien, France).

Table S1. Primary and secondary antibodies used for western blot. Dilutions are given for antibodies that are delivered in constituted form, and pg/ml for lyophilized antibodies

Target protein	Vendor	Catalog Nr	Raised in	Dilution
pERK 1/2	CellSignalling[1]	9101	Rabbit	1:7500
ERK 1/2	CellSignalling[1]	4695	Rabbit	1:10000
pSTAT 3	CellSignalling[1]	9145	Rabbit	1:1000
STAT 3	CellSignalling[1]	9139	Mouse	1:1000
Leptin	Peprotech[2]	500-P68	Rabbit	50pg/ml
Rabbit Ig	Dako[3]	P0448	Goat	1:2000
Mouse Ig	Dako[3]	P0161	Rabbit	1:2000

[1] Cell Signaling Technology, Inc., Danvers, USA [2] PeproTech, New Jersey, USA [3] DakoCytomation, Denmark
